# Hybrid Bone Substitute Containing Tricalcium Phosphate and Silver Modified Hydroxyapatite–Methylcellulose Granules

**DOI:** 10.3390/jfb15070196

**Published:** 2024-07-17

**Authors:** Joanna P. Czechowska, Annett Dorner-Reisel, Aneta Zima

**Affiliations:** 1 Faculty of Materials Science and Ceramics, AGH University of Krakow, 30 Mickiewicza Av., 30-059 Krakow, Poland; 2 Faculty of Mechanical Engineering, Schmalkalden University of Applied Sciences, 98574 Schmalkalden, Germany

**Keywords:** calcium phosphate, silver, hybrid

## Abstract

Despite years of extensive research, achieving the optimal properties for calcium phosphate-based biomaterials remains an ongoing challenge. Recently, ‘biomicroconcretes’ systems consisting of setting-phase-forming bone cement matrix and aggregates (granules/microspheres) have been developed and studied. However, further investigations are necessary to clarify the complex interplay between the synthesis, structure, and properties of these materials. This article focusses on the development and potential applications of hybrid biomaterials based on alpha-tricalcium phosphate (αTCP), hydroxyapatite (HA) and methylcellulose (MC) modified with silver (0.1 wt.% or 1.0 wt.%). The study presents the synthesis and characterization of silver-modified hybrid granules and seeks to determine the possibility and efficiency of incorporating these hybrid granules into αTCP-based biomicroconcretes. The αTCP and hydroxyapatite provide structural integrity and osteoconductivity, the presence of silver imparts antimicrobial properties, and MC allows for the self-assembling of granules. This combination creates an ideal environment for bone regeneration, while it potentially may prevent bacterial colonization and infection. The material’s chemical and phase composition, setting times, compressive strength, microstructure, chemical stability, and bioactive potential in simulated body fluid are systematically investigated. The results of the setting time measurements showed that both the size and the composition of granules (especially the hybrid nature) have an impact on the setting process of biomicroconcretes. The addition of silver resulted in prolonged setting times compared to the unmodified materials. Developed biomicroconcretes, despite exhibiting lower compressive strength compared to traditional calcium phosphate cements, fall within the range of human cancellous bone and demonstrate chemical stability and bioactive potential, indicating their suitability for bone substitution and regeneration. Further in vitro studies and in vivo assessments are needed to check the potential of these biomaterials in clinical applications.

## 1. Introduction

Biomaterials based on calcium phosphate are commonly used in biomedical applications, especially as hard tissue substitutes, as they demonstrate excellent biocompatibility and bioactivity. Calcium phosphate-based bone cements (CPCs) have emerged as prominent biomaterials for orthopedic applications. One advantage of chemically bonded bone cements over sintered calcium phosphate-based materials is their moldable form and setting properties. These cements undergo a setting reaction that transforms their powder and liquid components into a solid, cohesive matrix. The addition of a liquid phase triggers the setting process, resulting in cement hardening. By adjusting the powder-to-liquid ratio, setting time, and incorporating additives or modifiers, the mechanical and biological properties of the cement can be tailored. CPCs offer unique properties that make them highly suitable for bone regeneration and repair, including their resemblance to inorganic components of natural bone and their unique self-setting ability and resorb in vivo [1,2,3]. This versatile nature makes them highly valuable biomaterials. Despite years of extensive research and the introduction of multiple products to the market, achieving the optimal properties for calcium phosphate-based chemically bonded biomaterials remains an ongoing challenge. One of the main problems that researchers are facing is the low mechanical strength of calcium phosphate cements, which does not allow their use in load-bearing areas as well as their slow vascularization [1,2,3]. Various biocompatible additives such as polymeric fibers (e.g., poly (vinyl alcohol) or poly(l-lactic acid) fibers) were introduced to CPC formulations to enhance their mechanical properties [4,5,6]. Additionally, the rheological properties of CPCs often need to be optimized for 3D printing applications to reduce the filter-pressing of pastes [7,8]. Efforts are being made to modify these materials with antibacterial agents and drugs to prevent the development of chronic inflammatory conditions [9,10].

CPCs are composed mainly of calcium phosphates such as α-tricalcium phosphate (αTCP), dicalcium phosphate (DCP) and hydroxyapatite (HAp). Cements based on αTCP are especially interesting as bone-regenerating materials because they form calcium-deficient hydroxyapatite (CDHA), which is similar to the apatite found in bone [11]. This reaction was first described by Monma and Kanazawa [12,13] and afterward, numerous αTCP-based biomaterials were developed [14,15]. The osteoconductive properties of αTCP facilitate the creation of new bone by providing a scaffold for cellular attachment, proliferation, and differentiation. There are numerous prospects for development in αTCP applications, particularly in the production of composites. Therefore, further investigations are necessary to clarify the complex interplay between the synthesis, structure, and properties of αTCP-based materials. Recently, many scientists have focused on complex chemically bonded biomaterials, which include components responsible for the setting process, and also additional constituents such as granules, microspheres, or nanoparticles. The term ‘biomicroconcretes’ has been established in the literature to describe systems consisting of setting-phase-forming bone cement matrix and aggregates (granules/microspheres) [16]. In biomicroconcretes, these aggregates may serve as bioactive carriers for drugs or biologically active substances (e.g., antimicrobial agents, growth factors, etc.) [17,18,19]. Depending on the composition, granule/microspheres may act as bioresorbable, pore-forming components or, quite the opposite, they may provide mechanical support, ultimately influencing the properties of biomaterials [20,21]. For instance, H. Li et al. [22] have produced composite pellets consisting of poly(lactic-co-glycolic acid) microspheres and calcium phosphate cement. The presence of microspheres not only enhances the compressive strength but also creates in situ pores after the degradation of microspheres and prevents cells from dropping off materials. Calcium phosphate-based biomicroconcretes, despite being widely used, possess restricted antibacterial efficacy, which can hinder their effectiveness in environments susceptible to infection. Scientists have been recently trying to examined and addressed this concern. One approach can be material modification with antibacterial ions such as silver, gold or copper. Previous study demonstrated that multiphase αTCP-based cements, containing inter alia MgCHA, AgHA and calcite, enhanced cell viability [23,24] and silver-doped CPCs revealed antibacterial activity [25]. Another approach involves combining these materials with drugs, antibacterial agents, or nanoparticles (e.g., AuNPs, AgNPs) or the development of hybrid materials. The introduction of high amounts of antibacterial agents potentially may be a risk for human health. It is known that silver ions (Ag+) interact and strongly bind to albumins, metallothionein, and macroglobulins, but they are metabolized in all tissues except the brain and central nervous system. The maximum capacity of human blood to carry silver is not precisely known. On the other hand, the effects of silver nanoparticles (AgNPs) on human health are well documented and potentially cause toxic and harmful effects upon exposure [26,27]. Detailed discussions on the impact of silver ions on the body have been previously presented by A. B. Lansdown [28]. For bone applications, different types of silver salts, such as oxides, chlorides, sulfates, or phosphates in concentrations of 0.05% to 1% by weight, showed biocompatibility and elicited minimal foreign body reactions [29,30].

Hybrid biomaterials have recently been the subject of increasing interest. According to the International Union of Pure and Applied Chemistry (IUPAC), hybrid materials are composed of an intimate mixture of inorganic or organic components or both types of constituents, usually interpenetrating on scales of less than 1 μm [31]. In materials science hybrid materials typically refer to composites consisting of two or more distinct types of materials, such as polymers, ceramics, metals, or nanoparticles, combined at the nanoscale or microscale level to achieve enhanced and sometimes unexpected properties or functionalities [32]. Hybrids are usually classified into two groups based on the binding forces between their components: (a) class 1 materials with weak interactions (such as van der Waals, electrostatic, or hydrogen bonds) and (b) class 2 materials with strong interactions such as covalent or ionic–covalent bonds [33,34]. Recently, materials containing hybrid-type granules on the basis of chitosan and hydroxyapatite were developed and examined [35]. In these kinds of systems, the combination of synthetic and natural components helps to create scaffolds that mimic the properties of native bone tissue. Czechowska et al. [36] studied calcium phosphate-based bone cements containing HAp-chitosan granules modified with gold nanoparticles (AuNPs). Such materials revealed both in vitro bioactivity and antibacterial action against *E. coli*, *S. epidermidis* and *S. aureus*. Methylcellulose was also shown to be useful as a delivery vehicle for active pharmaceutical ingredients (APIs) [37,38]. Using both amphiphilic and thermoresponsive properties of methylcellulose, composite particles with the methylcellulose matrix embedded with API nanocrystals were developed [39]. Also, the methylcellulose-based hydrogel designed for the release of silver nanoparticles via an intelligent, pH-activated mechanism was proposed by Bonetti et al. [38]. The possibility of obtaining silver nanoparticles in methylcellulose solution through reduction and stabilization by a polymer has been previously shown, among others, by Bhui et al. [40] and Nishikawa et al. [41]. Furthermore, a preliminary study on self-setting granules composed of methylcellulose and hydroxyapatite indicates that they hold promise as a component of biomicroconcretes [42].

This article aims to explore the potential of composites for bone substitution and regeneration, specifically focusing on alpha-tricalcium phosphate and silver-modified, self-assembling hybrid granules. αTCP and hydroxyapatite provide structural integrity and osteoconductivity, silver imparts antimicrobial properties, and MC allows for the self-assembling of granules and controlled release capabilities. These hybrid granules consisting of hydroxyapatite and methylcellulose seem to be promising candidates for bone substitution and regeneration due to their set of unique properties. However, the possibility and efficiency of incorporating hybrid granules into calcium phosphate-based chemically bonded biomaterials still needs to be checked and the interplay between their structure and properties needs to be clarified. The study seeks to shed light on the development and potential applications of these hybrid bone substitutes, presenting them as promising candidates for bone substitution and regeneration in the field of bone tissue engineering.

## 2. Materials and Methods

### 2.1. αTCP

The αTCP powder was synthesized by the wet chemical method, according to the procedure described previously [36]. Briefly, the αTCP powder was synthesized using chemically pure grade Ca(OH)_2_ and an 85 wt.% solution of H_3_PO_4_ (POCH, Gliwice, Poland) as reagents. First, the Ca(OH)_2_ suspension in double-distilled water was prepared using a stirrer and then the H_3_PO_4_ acid solution was added dropwise to the suspension. The precipitate that formed as a result of the reaction was left for maturation. Afterward, the precipitate was dried, sintered at 1300 °C, ground in an attritor mill and sieved (<0.063 mm).

### 2.2. Hybrid Granules

Self-assembling hybrid, non-modified (GH) and silver-modified hydroxyapatite/methyllcelullose-based granules (AgGH) were synthesized using the wet chemical method, described previously by Czechowska et al. [42]. Briefly, phosphoric acid was introduced into a silver-containing methylcellulose solution and the mixture was added dropwise to a suspension containing calcium ions. The pH value during synthesis was stabilized at ~11. After this, the synthesis the suspension was aged (~24 h) and decanted. Precipitates were washed with distilled water, centrifuged and frozen. Subsequently, the materials were defrosted. Hybrid granules self-formed, and then they were dried (<40 °C) and sieved. Silver-modified hybrid granules containing 0.1 wt.% (AgGH-1) or 1.0 wt.% (AgGH-2) of silver were produced. In hybrid granules, the hydroxyapatite-to-methylcellulose ratio equaled 4:1. Chemically pure grade Ca(OH)_2_ (≥99.5%, Merck, Rahway, NJ USA), phosphoric acid (85.0%, Avantor Performance Materials, Gliwice, Poland), methylcellulose (Sigma-Aldrich, Poznań, Poland; viscosity: 4200 mPa∙s) and AgNO_3_ (Chempur, Piekary Śląskie, Poland) were used as reagents. SEM images of granules are presented in Figure S1 (Supplement).

### 2.3. Biomicroconcretes

The initial solid phases of materials were obtained by mixing the α-tricalcium phosphate powder with hybrid granules in a ratio of 3:2, respectively. To ensure the homogeneous distribution of the components of the solid phase, the powder batches were prepared by mixing αTCP powder with the granules, using a spatula. Two fractions of granules of sizes between 300 and 400 µm (S-small) and 400 and 600 µm (M-medium) were used. To produce the biomicroconcrete, the liquid phase was added and the components were stirred (~40–60 s) to produce a setting paste. Moreover, 0.5 wt.% methylcellulose solution in a 2.0 wt.% solution of Na_2_HPO_4_ was used as the liquid phase (Table 1). The components were mixed together in a liquid-to-powder ratio (L/P) equal to 0.64 g/g. Each material comes from a single batch. To produce samples of a desired shape and size, the modulable pastes were placed into Teflon molds and allowed to set. When needed, a material containing only αTCP in the powder phase was used as a control.

### 2.4. Chemical and Phase Composition

The crystalline phases of the initial powders, granules and set and hardened biomaterials were analyzed via powder X-ray diffraction with CuKα radiation, using a D2Phaser diffractometer, (Bruker, Karlsruhe, Germany). Measurements were conducted within the 2θ range from 10° to 60°. Each measurement was repeated three times. The phases were identified by comparing the experimental X-ray diffractograms with the International Centre for Diffraction Data—Joint Committee on Powder Diffraction Standards. HA (01-070-0798), αTCP (00-009-0348). Phase quantifications were carried out via the Rietveld refinement practical powder diffraction pattern analysis applying TOPAS. 

FT-IR spectra were obtained using Vertex 70v (Bruker, Germany). The frequency range was 400–4000 cm^−1^. The X-ray fluorescence method (XRF) using a WDXRF Axios Max (PANalytical, Malvern, UK) spectrometer (4 kW Rh source) was utilized.

### 2.5. Setting Times

The setting times of cement pastes were determined via Gilmore needles, according to the ASTM C266-08 standard [43]. The Gillmore apparatus consists of two needles with a flat cylindrical end. The initial setting needle possesses a diameter of 2.12 mm and weighs 113 g, whereas the final setting needle has a diameter of 1.06 mm and a weight of 453.6 g. During the measurements, the cement paste was placed in a mold and leveled. Then, the needles were positioned on the surface of the paste, released, and allowed to enter the paste. The initial setting time referred to the moment when the first testing needle no longer left a noticeable mark on the cement paste and the final setting time was the point in time when the second test needle was unable to penetrate the paste. All experiments were conducted at a temperature of 23 ± 2 °C. Measurements were repeated 6 times and the average value was calculated.

### 2.6. Compressive Strength

The compressive strength was tested using a universal material testing machine (Instron 3345). The crosshead displacement rate was 1.0 mm/min. For mechanical testing, cylindrical samples were prepared (6 mm in diameter, 12 mm in height). The compressive strength values were presented as the average of ten measurements ± standard deviation (SD). Differences in compressive strength were analyzed with one-way ANOVA with post hoc Tukey HSD (honestly significant difference) test (the mark (*) means statistically significant difference between the results).

### 2.7. Microstructure

The microstructures of fractured materials were observed using a scanning electron microscope (Nova NanoSem 200, FEI Company, Hillsboro, OR, USA; JEOL JSM 5400, Tokyo, Japan), using a lower secondary electron detector. To determine chemical compositions in microareas, an energy-dispersive X-ray spectroscopy (EDX) was applied. Before testing, the materials were coated with a thin layer of conductive carbon.

### 2.8. Chemical Stability and Bioactive Potential in SBF

The chemical stability of the biomicroconcretes were assessed in vitro in simulated body fluid (SBF). The applied SBF has ion concentrations nearly equivalent to that of human blood plasma (Ca^2+^ 2.5, Mg^2+^ 1.5, Na^+^ 142.0, K^+^ 5.0 mM, HPO_4_^2−^ 1.0, HCO_3_^−^ 4.2, Cl^−^ 147.8, SO_4_^2−^ 0.5 mM) and with a pH of ~7.40. The cylindrical samples (12 mm in diameter, 4 mm high) were incubated in SBF (40 mL) for 14 days (37 °C). The chemical stability was evaluated via a measurement of pH, using a pH/conduct-meter (Hanna H198129 Combo, Hanna Instruments, Smithfield, RI, USA). Results were presented as the average value of three measurements. Previously, it has been shown that the in vivo formation of an apatite layer on bioactive materials can be reproduced in a protein-free and acellular simulated body fluid [44]. To assess the bioactive potential of biomaterials, the SEM observation of their surfaces before and after storage in SBF were done (Nova NanoSem 200, JEOL JSM 5400).

## 3. Results

### 3.1. Chemical and Phase Composition

**Figure 1:**
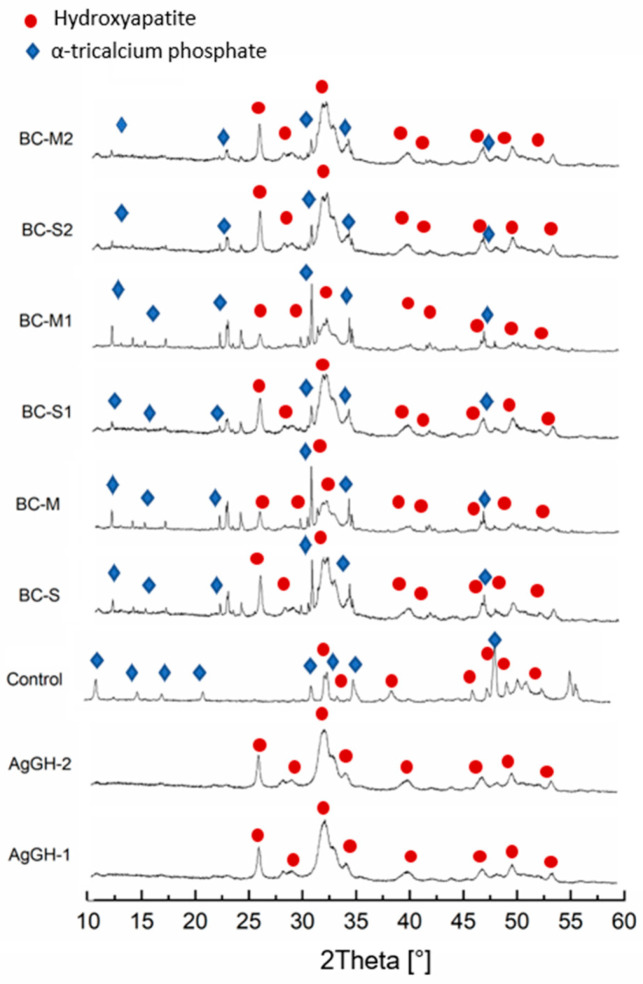
X-ray diffractograms of examined materials.

The chemical and phase composition of biomaterials, especially in the case of hybrids, is crucial for evaluating and optimizing their properties for specific applications. The XRF measurement confirmed that produced granules contain 0.11 wt.% (AgGH-1) and 0.93 wt.% (AgGH-2) of silver. The results of XRD measurements show that the granules are composed of hydroxyapatite as the only crystalline phase, whereas the set materials are composed of hydroxyapatite and α-tricalcium phosphate (Figure 1). No additional, undesirable crystalline phases were detected via XRD (Table 2).

XRD-detected hydroxyapatite is connected to both hydroxyapatite present in the granules and calcium-deficient hydroxyapatite from TCP hydrolysis. The reflexes of the hydroxyapatite phase exhibited broadening, indicating poor crystallinity in the granules (Figure 1). This also suggests the presence of poorly crystalline hydroxyapatite resulting from the αTCP setting process. The hydrolysis of αTCP leads to the formation of calcium-deficient hydroxyapatite (CDHA) with a Ca/P-ratio of c.a. 1.5, according to Equation (1) [45,46]

3Ca_3_(PO_4_)_2_ + H_2_O→Ca_9_(PO_4_)_5_(HPO_4_)OH (1)

αTCP +H_2_O → calcium-deficient hydroxyapatite

On the other hand, the results of Hurle et al. [47] suggests also the formation of an amorphous phase during the conversion of αTCP into CDHA, which may hinder the quantitative assessment of phase composition. It is well known that the HAp seeding in the solid phase of cements and presence of phosphate salts in the liquid phase accelerates th αTCP setting reaction. Thus, in our studies, the presence of hydroxyapatite in hydroxyapatite–methylcellulose granules as well as the application of Na_2_HPO_4_ as the liquid phase shifted the reaction toward the formation of calcium-deficient hydroxyapatite (Equation (1)). In previous research conducted by Pańtak et al. [48] and Dziadek et al. [16], the application of αTCP and hybrid HAp/chitosan granules resulted in just a modest increase (up to 10 wt.%) in the amount of the hydroxyapatite phase after setting in air. In the case of developed biomicroconcretes, a noticeable difference in composition between set, silver-modified and non-modified materials was observed (Table 2). The presence of silver favored the process of αTCP hydrolysis to calcium-deficient hydroxyapatite. Furthermore, the quantitative XRD analysis showed that the amount of hydroxyapatite in biomicroconcretes increased with increasing silver content (BC-M < BC-M1 < BC-M2). The inverse relationship can be observed for the size of granules, the bigger size the smaller amount of CDHA in biomicroconcretes (e.g., BC-S > BC-M; BC-S1 > BC-M1). The obtained results indicate that silver-modified materials favor αTCP hydrolysis to calcium-deficient hydroxyapatite. Explaining this phenomenon in such a complex system is challenging. However, the observed effect is likely associated with the chemical interactions between the components, facilitated by the hybrid nature of the granules. Chemical interactions may occur between the silver and both methylcellulose and hydroxyapatite, including (1) the formation of silver nanoparticles in the methylcellulose matrix [41,49] and/or (2) the partial substitution of calcium ions by silver ions in the hydroxyapatite structure. Both processes affect the structure and solubility of materials and therefore subsequently affect the hydrolysis of αTCP to calcium-deficient hydroxyapatite.

**Figure 2:**
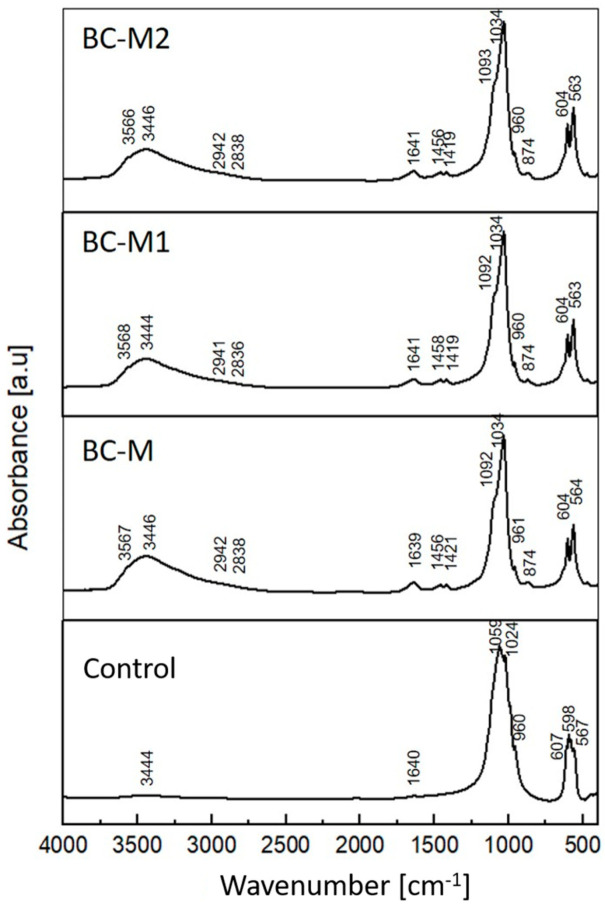
Fourier-transform infrared spectroscopy spectra of hardened biomaterials 7 days after setting in air.

The Fourier-transform infrared (FTIR) spectroscopy spectra of all set and hardened materials were similar (Figure 2). The strongest bands assigned to calcium phosphates were centered at 1034 cm^–1^ and around 1092 cm^−1^ and were assigned to anti-symmetric stretching modes of P-O. The typical bands corresponding to the carbonate groups are located around 874, 1419 and 1458 cm^−1^, indicating the presence of B-type carbonated hydroxyapatite in the materials. The carbonate ions were probably introduced to the hydroxyapatite structure during the synthesis. Bands at around 563 and 604 cm^–1^ were assigned to triply degenerate the P–O–P bending mode. The wide band at around 3446 cm^−1^ is connected to the vibratory stretching of the OH group of hydroxyapatites and the band at 1641 cm^−1^ is attributed to adsorbed water molecules and overlaps with the band assigned to C-O carbonyl stretching from the glucose in the methylcellulose. Methylcellulose possessed characteristic absorption bands at 1460, 1380, 1320 and 950 cm^−1^ attributed to the C−H stretching of CH_2_ and CH_3_ groups, as well as the 1110 cm^−1^ band assigned to C−O−C stretching within an anhydroglucose ring [50]. Also, bands connected to O-H stretching vibrations at around 3459 cm^−1^, C-H stretching at 2932 and 2843 cm^−1^ and C-O carbonyl stretching have been previously reported [51,52]. Some of these bands, such as those at 2942 cm^−1^ and 2838 cm^−1^, overlapped with bands assigned to calcium phosphates and were hardly visible due to the low concentration of the polymer in the materials.

### 3.2. Setting Times

The measurement of setting times is a crucial aspect in assessing the properties of chemically bonded materials, providing valuable information about their workability and strength development over time. Setting times measured for biomicroconcretes ranged from 8 to 14 min (initial) and 22 to 35 min (final) (Table 3). The initial setting time indicated the time available for placing the material into a bone void. Numerous records highlight that the αTCP setting process is initially controlled by a surface reaction, which depends mainly on the powdered dissolution [45,46,47]. This setting reaction creates a calcium phosphate precipitate and induces the hardening of the cement. Single-component αTCP cement (control) set within 9 ± 1 min (tf). However, some of the previous studies also demonstrated that the setting time obtained for chemically bonded biomaterials containing granules and a microsphere is longer than without the additives [34,36]. Furthermore, our research indicated also that the addition of silver to the granules resulted in a prolonged setting process (tf: 28–32 min) compared to the unmodified materials (tf: 22–23 min). Obtained values are slightly longer than recommended in the literature but still acceptable. The differences in setting times between the materials containing granules of different sizes and varying amounts of silver were negligible.

### 3.3. Microstructure

**Figure 3:**
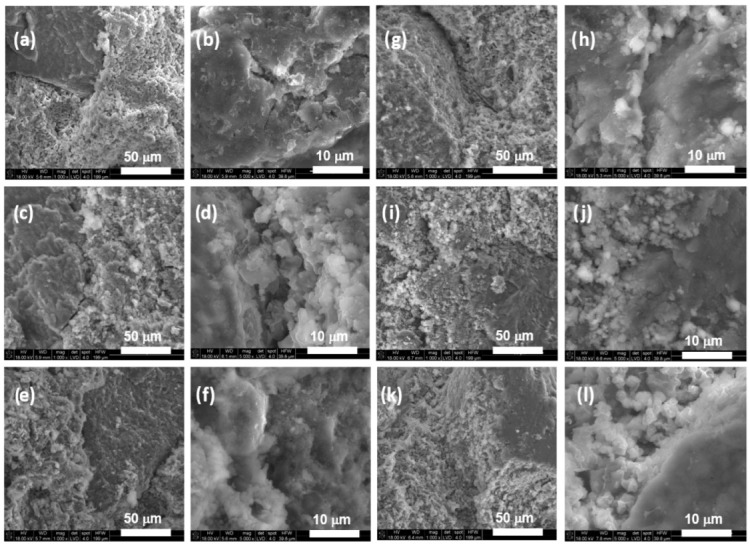
SEM microphotographs of biomaterials cross-sections: BC-S (**a**,**b**), BC-M (**c**,**d**), BC-S1 (**e**,**f**), BC-M1 (**g**,**h**), BC-S2 (**i**,**j**), BC-M2 (**k**,**l**) (mag. 1000× and 5000×, respectively).

**Figure 4:**
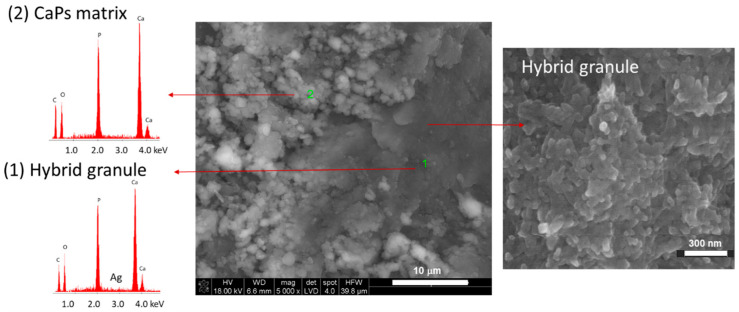
SEM microphotographs of BC-S2 cross-section with chemical analysis in microareas (EDS) of granules (1) and matrix (2).

SEM observations of the biomaterials’ cross-sections revealed that the matrix of the biomaterials is in general a structure of interconnected agglomerations of smaller crystals arranged in very dense packing with some visible micropores (Figure 3). The granules are of irregular shape and their morphology is more compact than the CaPs matrix. The nano-sized hydroxyapatite crystals and polymeric bridges are visible in hybrid materials (Figure 4).

Granules are embedded within the matrix, and good adhesion between both components can be noticed for all compositions. Chemical analysis in microareas revealed the presence of peaks typical for calcium phosphates. An absorption peak at 3 keV in the chemical analysis confirmed the presence of silver in hybrid granules (Figure 4).

### 3.4. Compressive Strength

**Figure 5:**
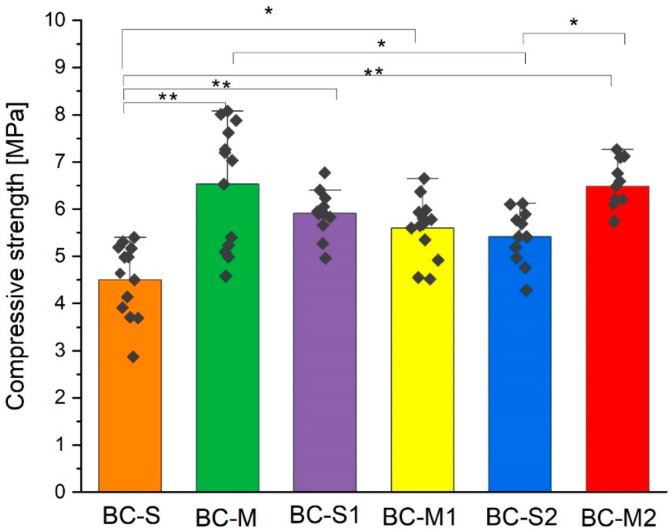
Compressive strength of biomicroconcretes 7 days after setting and hardening (n = 10) (* *p* < 0.05, ** *p* < 0.01).

Materials gain their mechanical strength during the setting process. While setting, calcium phosphate precipitates form, and the entanglement of crystals promotes an enhancement in the mechanical strength. In the literature, it is reported that TCP-based bone cements may possess a initial compressive strength up to 20–30 MPa, owing to its relatively compact microstructure after setting [13]. However, the mechanical properties of chemically bonded materials depend on numerous variables, such as the composition of the powder and liquid phase, the liquid-to-powder ratio (L/P), the method of cement preparation, and the adhesion between the constituents. The individual effects of these variables are difficult to isolate. The compressive strength of the obtained biomicroconretes ranged between 4.5 ± 0.8 MPa and 6.5 ± 1.3 MPa (Figure 5.) and was lower than for the control (13.1 ± 3.3 MPa). Similar compressive strength values, up to 6.54 MPa, were reported by Nezafati et al. [53] for tetra calcium phosphate-based bone cements enriched with silanated gelatin microspheres. Good adhesion between the components of biomicroconcretes (matrix and aggregate) is clearly evident in Figure 3 and Figure 4; thus, it does not appear to be a dominant factor in the mechanism leading to the decline in the mechanical strength of biomicroconcretes. However, it seems that the introduction of granules disrupted the continuous compact microstructure of the αTCP matrix and increased the amount of micropores in materials. Complex, chemically bonded systems, containing aggregates such as granules, pellets or microspheres are more difficult to homogenize, and thus, it is easier to introduce air bubbles into the cement paste during mixing. These trapped air pockets within the solidifying cement microstructure subsequently lead to the formation of micropores (Figure S2, Supplement). These phenomena are the main reasons for the compressive strength decrease. Statistically significant differences in compressive strength were observed for materials with different granule sizes, i.e., BC-M vs. BC-S and BC-M2 vs. BC-S2. Materials with medium (M)-sized granules possessed higher mechanical strength compared to small (S) granules. The compressive strength of specimens can be influenced by the matrix strength, the granule size, and the bond between the granules and the cement matrix. Differences in the mechanical strength of materials containing granules of various sizes may be due to subtle variations in the distribution of medium and small granules within the matrix, as well as differences in the specific surface area of the granules. The compressive strength of all biomicroconcretes falls within the range of human cancellous bone, which is typically between 2 and 12 MPa [54]. Our findings align with the work of Kamitakahara et al. [20], who indicated that an increased quantity of CDHA or octacalcium phosphate-based granules in the CPC formulation led to a significant decrease in compressive strength. Similar results were also obtained by Hasan et al. [55] on calcium phosphate bone cements containing bioglass-based microspheres. Hasan et al. have found that the compressive strength of complex formulations was lower compared to CPCs and decreased with an increasing content of granules.

### 3.5. Chemical Stability and Bioactive Potential

**Figure 6:**
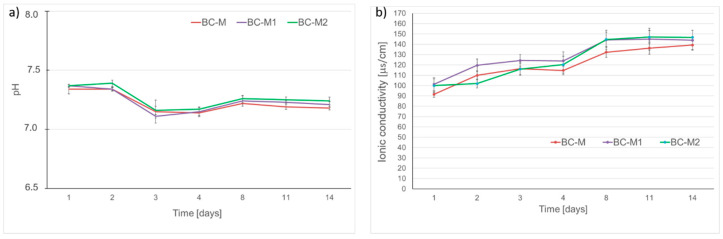
Chemical stability: pH vs. time of incubation in SBF (**a**), ionic conductivity vs. time of incubation in distilled water (**b**) (n = 3).

Biomaterial stability refers not only to mechanical durability (e.g., resistance to cracking) but also chemical stability, particularly in challenging biological environments. It is known that in vitro, the process of the dissolution of materials depends on the dissolution environment used [56]. As previously stated, some local chemical changes caused by implant material can influence the behavior of bone cells [57,58]. Therefore, the chemical stability of biomaterials was assessed by measuring changes in pH and ionic conductivity. The pH measurements showed only a slight decrease in pH of SBF from 7.4 to ~7.2 during the 14 days of incubation (Figure 6a). The pH level of the SBF solution surrounding the samples stayed near the physiological range.

Figure 6b, demonstrated a minor increase in ionic conductivity connected with the samples’ dissolution and ion release, up to 139 ± 4 µS/cm (BC-M), 144 ± 9 µS/cm (BC-M1), and 147 ± 6 µS/cm (BC-M2). In vitro studies on αTCP-based cements with a similar ion release profile conducted by Przekora et al. [59] did not display cytotoxicity.

**Figure 7:**
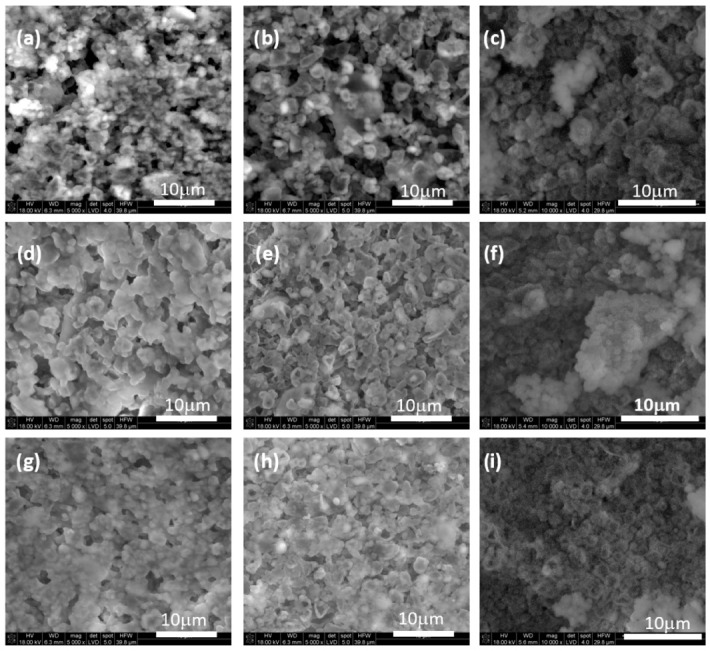
SEM microphotographs of biomicroconcrete surfaces before and after incubation in SBF; (**a**) BC-M before incubation, (**b**) BC-M after 7-day incubation, (**c**) BC-M after 14-day incubation in SBF; (**d**) BC-M1 before incubation, (**e**) BC-M1 after 7-day incubation, (**f**) BC-M1 after 14-day incubation in SBF; (**g**) BC-M2 before incubation, (**h**) BC-M2 after 7-day incubation, (**i**) BC-M2 after 14-day incubation in SBF.

After incubation in simulated body fluid (SBF), certain alterations in surface morphologies, associated with creation of an apatite layer, can be observed for all biomaterials (Figure 7). Kokubo et al. [44] proposed a mechanism by which hydroxyapatite in a simulated body fluid becomes negatively charged and attracts Ca^2+^ ions to form calcium-rich calcium phosphate. The accumulation of calcium ions leads to the formation of a positively charged surface, which facilitates binding with phosphate ions and produce amorphous calcium phosphate. This phase transforms over time into stable, bone-like apatite. If materials immersed in SBF develop the apatite layer on their surface within a period of 28 days, they can be considered potentially bioactive. In our study, as soon as 7 days of incubation pass, the layer evenly covers the surfaces of all incubated biomicroconcretes (Figure 7). Therefore, it appears that the presence of silver-modified hybrid granules has a positive impact on the bioactive potential of materials. Furthermore, this creates an ideal environment for bone regeneration, while it simultaneously may prevent bacterial colonization and infection due to the presence of silver. Conducted in vitro tests in SBF revealed that the examined materials possess bioactive potential. However, further in vivo studies are necessary to confirm this hypothesis.

## 4. Discussion

Hybrid materials have gained significant attention in the field of bone tissue engineering due to their unique combination of properties derived from different components. These studies were carried out to clarify the complex interplay between the synthesis, structure, and properties of αTCP-based biomicroconcretes containing self-assembling, silver-modified hydroxyapatite–methylcellulose-based granules. Two amounts of silver, namely 0.11 wt.% or 0.93 wt.%, were successfully incorporated into hybrid granules. This combination potentially will support bone regeneration, while simultaneously preventing bacterial colonization and infection. The research checked the impact of hybrid granules and silver modifications on the setting process and physicochemical properties of the materials. X-ray diffractometry revealed that materials were composed of two crystalline phases: hydroxyapatite and α-tricalcium phosphate. It was demonstrated that the presence of silver favored the process of αTCP hydrolysis to calcium-deficient hydroxyapatite and the amount of hydroxyapatite in biomicroconcretes increased with increasing silver content. Fourier-transform infrared spectroscopy confirmed the existence of vibrations of groups corresponding to calcium phosphates, along with specific bands associated with methylcellulose. The results of the setting time measurements showed that both the size and the composition of granules have an impact on the setting process of biomicroconcretes. The addition of silver to the hybrid granules resulted in the prolongation of setting times from 8 ± 1 min to 13 ± 1 min (initial) and from 22 ± 2 min to 32 ± 2 min (final). This effect is associated with the chemical interactions between the components, facilitated by their hybrid nature. These chemical interactions may include the formation of silver nanoparticles in a methylcellulose matrix and the partial substitution of Ca^2+^ by Ag^+^ in the hydroxyapatite structure, which impact the solubility and subsequently affect the hydrolysis of αTCP. SEM observations of set materials demonstrated good adhesion of the components on the matrix/granule interphase. The compressive strength of all materials (4.5 ± 0.8 MPa–6.5 ± 1.3 MPa) falls within the range of human cancellous bone. As complex, chemically bonded systems containing aggregates such as granules, pellets or microspheres are generally more difficult to homogenize, some decrease in mechanical strength compared to the control (13.1 ± 3.3 MPa) was visible and expected. Statistically significant differences in compressive strength for biomicroconcretes were observed in favor of materials with larger granules. All biomaterials were found to be chemically stable in conditions simulating the environment of a living organism. Only small fluctuations in pH of SBF were noticed during the incubation of samples for 14 days. The dissolution–precipitation process in simulated body fluid led to the formation of an apatite layer on the materials’ surfaces, which indicated their bioactive potential. 

To conclude, self-assembling silver-modified methylcellulose/hydroxyapatite-based hybrid granules seems to be a promising candidate for bone substitution. Adding hybrid granules to surgically handy chemically bonded biomaterials shows potential for improving antibacterial features without compromising the desired qualities for bone tissue engineering. As silver may be harmful not only to bacterial strains but also for bone cells, additional research, including antibacterial tests and cytocompatibility testing, is needed to confirm the effectiveness of these biomicroconcretes in clinical use.

## Figures and Tables

**Table 1:** Compositions of the studied materials.

Material	Solid Phase *		Liquid Phase	L/P [g/g]
	Granules	Powder		
BC-S	GH 300–400 µm	αTCP	0.5 wt.% methylcellulose in 2.0 wt.% Na_2_HPO_4_	0.64
BC-M	GH 400–600 µm			
BC-S1	AgGH-1 (0.1 wt.% Ag) 300–400 µm	αTCP	0.5 wt.% methylcellulose in 2.0 wt.% Na_2_HPO_4_	0.64
BC-M1	AgGH-1 (0.1 wt.% Ag) 400–600 µm			
BC- S2	AgGH-2 (1.0 wt.% Ag) 300–400 µm	αTCP	0.5 wt.% methylcellulose in 2.0 wt.% Na_2_HPO_4_	0.64
BC-M2	AgGH-2 (1.0 wt.% Ag) 400–600 µm			
Control	--	αTCP	0.5 wt.% methylcellulose in 2.0 wt.% Na_2_HPO_4_	0.48

* Solid phase: αTCP powder-to-granule ratio is equal to 3:2.

**Table 2:** Phase composition of set and hardened cement bodies (n = 3).

Material	Hydroxyapatite [wt.%]	αTCP [wt.%]
BC-S	73 ± 5	27 ± 5
BC-M	55 ± 4	45 ± 4
BC-S1	89 ± 1	11 ± 1
BC-M1	62 ± 4	38 ± 4
BC- S2	92 ± 1	8 ± 1
BC-M2	93 ± 1	7 ± 1
Control	40 ± 4	60 ± 4

**Table 3:** Setting times of the materials (n = 6).

Cement	Initial t_i_ [min]	Final t_f_ [min]
BC-S	8 ± 1	22 ± 2
BC-M	10 ± 1	24 ± 2
BC-S1	13 ± 1	28 ± 2
BC-M1	14 ± 1	29 ± 2
BC-S2	12 ± 1	32 ± 2
BC-M2	13 ± 1	30 ± 3
Control	4 ± 1	9 ± 1

## Data Availability

The original contributions presented in the study are included in the article; further inquiries can be directed to the corresponding authors.

## Supplementary Materials

The following supporting information can be downloaded at: https://www.mdpi.com/article/10.3390/jfb15070196/s1. Figure S1: SEM microphotographs of granules: (a) AgGH-1 and (b) AgGH-2. (mag. 350× and 5000×, respectively). Figure S2: Cumulative porosity MIP curves of Control and BC-S1 biomicroconcrete.

## References

[1] Vezenkova A., Locs J. (2022). Sudoku of porous, injectable calcium phosphate cements–Path to osteoinductivity. Bioact. Mater..

[2] Wang X.H., Jia S.J., Hao D.J. (2020). Advances in the modification of injectable calcium-phosphate-based bone cements for clinical application. Chin. Med. J..

[3] Demir-Oğuz Ö., Boccaccini A.R., Loca D. (2023). Injectable bone cements: What benefits the combination of calcium phosphates and bioactive glasses could bring?. Bioact. Mater..

[4] Zuo Y., Yang F., Wolke J.G., Li Y., Jansen J.A. (2010). Incorporation of biodegradable electrospun fibers into calcium phosphate cement for bone regeneration. Acta Biomater..

[5] Kucko N.W., de Lacerda Schickert S., Sobral Marques T., Herber R.P., Van den Beuken J.J., Zuo Y., Leeuwenburgh S.C. (2019). Tough and osteocompatible calcium phosphate cements reinforced with poly (vinyl alcohol) fibers. ACS Biomater. Sci. Eng..

[6] Paknahad A., Goudarzi M., Kucko N.W., Leeuwenburgh S.C., Sluys L.J. (2021). Calcium phosphate cement reinforced with poly (vinyl alcohol) fibers: An experimental and numerical failure analysis. Acta Biomater..

[7] Weichhold J., Pfeiffle M., Kade J.C., Hurle K., Gbureck U. (2023). Aqueous calcium phosphate cement inks for 3D printing. Adv. Eng. Mater..

[8] Xiong X., Chen Y., Yuan R., Qiu G., Weir M.D., Xu H.H.K., Liu J., Ruan J., Chang X., Qu S. (2022). 3D-printed mechanically strong calcium phosphate cement scaffold with metformin/stem cell-encapsulating alginate microbeads for bone tissue engineering. J. Bionic Eng..

[9] Huang S.M., Chen W.C., Wu C.C., Liu S.M., Ko C.L., Chen J.C., Shih C.J. (2023). Synergistic effect of drug/antibiotic-impregnated micro/nanohybrid mesoporous bioactive glass/calcium phosphate composite bone cement on antibacterial and osteoconductive activities. Biomater. Adv..

[10] Qiu G., Huang M., Liu J., Wang P., Schneider A., Ren K., Oates T.W., Weir M.D., Xu H.H.K., Zhao L. (2021). Antibacterial calcium phosphate cement with human periodontal ligament stem cell-microbeads to enhance bone regeneration and combat infection. J. Tissue Eng. Regen. Med..

[11] Pina S., Olhero S.M., Gheduzzi S., Miles A.W., Ferreira J.M.F. (2009). Influence of setting liquid composition and liquid-to-powder ratio on properties of a Mg-substituted calcium phosphate cement. Acta Biomater..

[12] Monma H., Kanazawa T. (1976). The hydration of α-tricalcium phosphate. J. Ceram. Assoc..

[13] Tronco M.C., Cassel J.B., Dos Santos L.A. (2022). α-TCP-based calcium phosphate cements: A critical review. Acta Biomater..

[14] Siek D., Ślósarczyk A., Przekora A., Belcarz A., Zima A., Ginalska G., Czechowska J. (2017). Evaluation of antibacterial activity and cytocompatibility of α-TCP based bone cements with silver-doped hydroxyapatite and CaCO_3_. Ceram. Int..

[15] Fernández E., Vlad M.D., Hamcerencu M., Darie A., Torres R., Lopez J. (2005). Effect of iron on the setting properties of α-TCP bone cements. J. Mater. Sci..

[16] Dziadek M., Zima A., Cichoń E., Czechowska J., Ślósarczyk A. (2020). Biomicroconcretes based on the hybrid HAp/CTS granules, α-TCP and pectins as a novel injectable bone substitutes. Mater. Lett..

[17] Wu S., Lei L., Bao C., Liu J., Weir M.D., Ren K., Schneider A., Oates T.W., Liu J., Xu H.H. (2021). An injectable and antibacterial calcium phosphate scaffold inhibiting Staphylococcus aureus and supporting stem cells for bone regeneration. Mater. Sci. Eng. C.

[18] Hu M.H., Chu P.Y., Huang S.M., Shih B.S., Ko C.L., Hu J.J., Chen W.C. (2022). Injectability, processability, drug loading, and antibacterial activity of gentamicin-impregnated mesoporous bioactive glass composite calcium phosphate bone cement in vitro. Biomimetics.

[19] Nuzulia N.A., Mart T., Ahmed I., Sari Y.W. (2024). The Use of Microspheres for Cancer Embolization Therapy: Recent Advancements and Prospective. ACS Biomater. Sci. Eng..

[20] Kamitakahara M., Asahara K., Matsubara H. (2022). Calcium phosphate cements comprising spherical porous calcium phosphate granules: Synthesis, structure, and properties. J. Asian Ceram. Soc..

[21] Meng D., Dong L., Wen Y., Xie Q. (2015). Effects of adding resorbable chitosan microspheres to calcium phosphate cements for bone regeneration. Mater. Sci. Eng. C.

[22] Li H., Li J., Ye J. (2016). Construction and properties of poly (lactic-co-glycolic acid)/calcium phosphate cement composite pellets with microspheres-in-pellet structure for bone repair. Ceram. Int..

[23] Zima A., Czechowska J., Siek D., Olkowski R., Noga M., Lewandowska-Szumieł M., Ślósarczyk A. (2017). How calcite and modified hydroxyapatite influence physicochemical properties and cytocompatibility of alpha-TCP based bone cements. J. Mater. Sci. Mater. Med..

[24] Oh S.A., Lee G.S., Park J.H., Kim H.W. (2010). Osteoclastic cell behaviors affected by the α-tricalcium phosphate based bone cements. J. Mater. Sci. Mater. Med..

[25] Rau J.V., Fosca M., Graziani V., Egorov A.A., Zobkov Y.V., Fedotov A.Y., Ortenzi M., Caminiti R., Baranchikov A.E., Komlev V.S. (2016). Silver-doped calcium phosphate bone cements with antibacterial properties. J. Funct. Biomater..

[26] Li Y., Cummins E. (2020). Hazard characterization of silver nanoparticles for human exposure routes. J. Environ. Sci. Health Part A.

[27] Tortella G.R., Rubilar O., Durán N., Diez M.C., Martínez M., Parada J., Seabra A.B. (2020). Silver nanoparticles: Toxicity in model organisms as an overview of its hazard for human health and the environment. J. Hazard. Mater..

[28] Lansdown A.B. (2010). A pharmacological and toxicological profile of silver as an antimicrobial agent in medical devices. Adv. Pharmacol. Pharm. Sci..

[29] Spadaro J.A., Webster D.A., Becker R.O. (1979). O. Silver polymethyl methacrylate antibacterial bone cement. Clin. Orthop. Relat. Res..

[30] Liu H., Li P., Tang Z., Liu H., Zhang R., Ge J., Yang H., Ni X., Lin X., Yang L. (2023). Study on injectable silver-incorporated calcium phosphate composite with enhanced antibacterial and biomechanical properties for fighting bone cement-associated infections. Colloids Surf. B Biointerfaces.

[31] (2007). Definitions of terms relating to the structure and processing of sols, gels, networks, and inorganic-organic hybrid materials (IUPAC Recommendations 2007). PAC.

[32] Mir S.H., Nagahara L.A., Thundat T., Mokarian-Tabari P., Furukawa H., Khosla A. (2018). Organic-inorganic hybrid functional materials: An integrated platform for applied technologies. J. Electrochem. Soc..

[33] Sanchez C., Ribot F., Lebeau B. (1999). Molecular design of hybrid organic-inorganic nanocomposites synthesized via sol-gel chemistry. J. Mater. Chem..

[34] Park W., Shin H., Choi B., Rhim W.K., Na K., Han D.K. (2020). Advanced hybrid nanomaterials for biomedical applications. Prog. Mater. Sci..

[35] Zima A. (2018). Hydroxyapatite-chitosan based bioactive hybrid biomaterials with improved mechanical strength. Spectrochim. Acta Part A Mol. Biomol. Spectrosc..

[36] Czechowska J., Cichoń E., Belcarz A., Ślósarczyk A., Zima A. (2021). Effect of gold nanoparticles and silicon on the bioactivity and antibacterial properties of hydroxyapatite/chitosan/tricalcium phosphate-based biomicroconcretes. Materials.

[37] Nunes M.R., de Souza Maguerroski Castilho M., de Lima Veeck A.P., da Rosa C.G., Noronha C.M., Maciel M.V.O.B., Barreto P.M. (2018). Antioxidant and antimicrobial methylcellulose films containing Lippia alba extract and silver nanoparticles. Carbohydr. Polym..

[38] Bonetti L., Fiorati A., D’Agostino A., Pelacani C.M., Chiesa R., Farè S., De Nardo L. (2022). Smart methylcellulose hydrogels for pH-triggered delivery of silver nanoparticles. Gels.

[39] Chen L.H., Doyle P.S. (2021). Design and use of a thermogelling methylcellulose nanoemulsion to formulate nanocrystalline oral dosage forms. Adv. Mater..

[40] Bhui D.K., Pyne S., Sarkar P., Bar H., Sahoo G.P., Misra A. (2011). Temperature controlled synthesis of silver nanostructures of variable morphologies in aqueous methyl cellulose matrix. J. Mol. Liq..

[41] Nishikawa H., Nakata E., Nakano S., Nakajima T., Morii T. (2020). Influence of polymer molecular weight on the properties of in situ synthesized silver–methylcellulose nanocomposite films with a CO_2_ laser. J. Mater. Sci..

[42] Czechowska J. (2021). Self-assembling, hybrid hydroxyapatite-methylcellulose granules, modified with nano-silver. Mater. Lett..

[43] (2021). Standard Test Method for Time Setting of Hydraulic-Cement Paste by Gillmore Needles in ASTM Annual Book of Standards.

[44] Kokubo T., Kim H.M., Kawashita M. (2003). Novel bioactive materials with different mechanical properties. Biomaterials.

[45] Fernandez E., Gil F.J., Ginebra M.P., Driessens F.C.M., Planell J.A., Best S.M. (1999). Calcium phosphate bone cements for clinical applications. Part I: Solution chemistry. J. Mater. Sci. Mater. Med..

[46] Carrodeguas R.G., De Aza S. (2011). α-Tricalcium phosphate: Synthesis, properties and biomedical applications. Acta Biomater..

[47] Hurle K., Neubauer J., Bohner M., Doebelin N., Goetz-Neunhoeffer F. (2014). Effect of amorphous phases during the hydraulic conversion of α-TCP into calcium-deficient hydroxyapatite. Acta Biomater..

[48] Pańtak P., Czechowska J.P., Zima A. (2023). The influence of silane coupling agents on the properties of α-TCP-based ceramic bone substitutes for orthopaedic applications. RSC Adv..

[49] Maity D., Mollick M.M.R., Mondal D., Bhowmick B., Bain M.K., Bankura K., Sarkar J., Acharya K., Chattopadhyay D. (2012). Synthesis of methylcellulose–silver nanocomposite and investigation of mechanical and antimicrobial properties. Carbohydr. Polym..

[50] Oliveira R.L., Vieira J.G., Barud H.S., Assunção R., Filho G.R., Ribeiro S.J.L., Messadeqq Y. (2015). Synthesis and characterization of methylcellulose produced from bacterial cellulose under heterogeneous condition. J. Braz. Chem. Soc..

[51] Rangelova N., Radev L., Nenkova S., Miranda Salvado I.M., Vas Fernandes M.H., Herzog M. (2011). Methylcellulose/SiO_2_ hybrids: Sol-gel preparation characterization by XRD, FTIR and AFM. Cent. Eur. J. Chem..

[52] Viera R.G., Rodrigues Filho G., de Assunção R.M., Meireles C.D.S., Vieira J.G., de Oliveira G.S. (2007). Synthesis and characterization of methylcellulose from sugar cane bagasse cellulose. Carbohydr. Polym..

[53] Nezafati N., Farokhi M., Heydari M., Hesaraki S., Nasab N.A. (2019). In vitro bioactivity and cytocompatablity of an injectable calcium phosphate cement/silanated gelatin microsphere composite bone cement. Compos. Part B Eng..

[54] Misch C.E., Qu Z., Bidez M.W. (1999). Mechanical properties of trabecular bone in the human mandible: Implications for dental implant treatment planning and surgical placement. J. Oral Maxillofac. Surg..

[55] Hasan M.L., Kim B., Padalhin A.R., Faruq O., Sultana T., Lee B.T. (2019). In vitro and in vivo evaluation of bioglass microspheres incorporated brushite cement for bone regeneration. Mater. Sci. Eng. C.

[56] Shah F.A., Brauer D.S., Wilson R.M., Hill R.G., Hing K.A. (2014). Influence of cell culture medium composition on in vitro dissolution behavior of a fluoride-containing bioactive glass. J. Biomed. Mater. Res. Part A.

[57] Zalewska J., Przekora A., Pałka K., Belcarz A. (2022). Gypsum-related compensation of ions uptake by highly porous hydroxyapatite ceramics–Consequences for osteoblasts growth and proliferation. Biomater. Adv..

[58] Bernhardt A., Schamel M., Gbureck U., Gelinsky M. (2017). Osteoclastic differentiation and resorption is modulated by bioactive metal ions Co^2+^, Cu^2+^ and Cr^3+^ incorporated into calcium phosphate bone cements. PLoS ONE.

[59] Przekora A., Czechowska J., Pijocha D., Ślósarczyk A., Ginalska G. (2014). Do novel cement-type biomaterials reveal ion reactivity that affects cell viability in vitro?. Open Life Sci..

