# Supplementary material for: Hybrid Bone Substitute Containing Tricalcium Phosphate and Silver Modified Hydroxyapatite–Methylcellulose Granules
Source: J Funct Biomater. 2024 Jul 17;15(7):196. doi: 10.3390/jfb15070196 (PMC11278312; doi:10.3390/jfb15070196)
Supplement: Supplementary file 1 [file jfb-15-00196-s001.zip › jfb-3081052-supplementary.pdf]

Supplementary data:

Morphology of granules was presented in Figure S1.

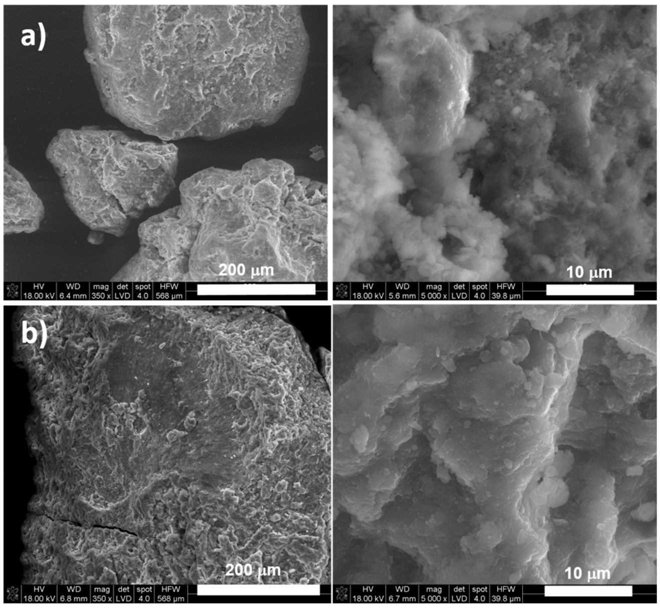

Figure S1. SEM microphotographs of granules: a) AgGH-1 and b) AgGH-2. (mag. 350x and 5000x, respectively).

The open porosity and the pore-size distribution of the Control material (without aggregate) and biomicroconcrete BC-S1 were compared using mercury porosimeter (AutoPore IV 9500). Open porosity and pore size distributions were generated from the pressure versus intrusion data using the Washburn equation.

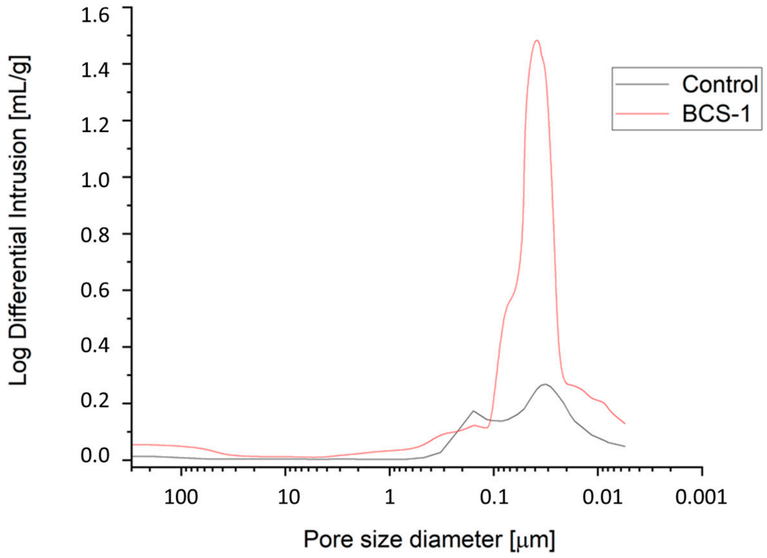

Figure S2. Cumulative porosity MIP curves of Control and BC-S1 biomicroconcrete.
